# Numerical Simulation Study on Impact Initiation on Shielded Charge Using Hypervelocity Composite-Structure Reactive Fragments

**DOI:** 10.3390/polym16081054

**Published:** 2024-04-11

**Authors:** Yongjin Lu, Bo Tan, Yanxia Li, Sheng Tan, Shixi Yang, Wensu Ji

**Affiliations:** 1 College of Weaponry Engineering, Naval University of Engineering, Wuhan 430033, China; d22382602@nue.edu.cn (Y.L.);; 2 Wuhan Vocational College of Foreign Language and Foreign Affair, Wuhan 430083, China

**Keywords:** PTFE/Al material, hypervelocity impact, shielded charge, detonation, impact energy release

## Abstract

In order to study the impact initiation process and mechanism of hypervelocity PTFE/Al composite structure reactive fragments on a shielded charge, first, an existing PTFE/Al reactive fragment hypervelocity collision experiment was numerically simulated using the SPH algorithm in ANSYS/AUTODYN 17.0 software. Then, the Lee–Tarver model was verified to describe the detonation reaction behavior and explosion damage effect of reactive materials. A numerical simulation analysis of the impact of two kinds of ultra-high-speed PTFE/Al composite-structure reactive fragments on a shielded charge was carried out using the SPH algorithm. These were steel-coated PTFE/Al and steel-semi-coated PTFE/Al fragments, and they were compared with the impact of steel fragments. The results indicate that the threshold velocities of the impact initiation of the two composite-structure reactive fragments on the shielded charge were both 2.6 km/s, while the threshold velocity of the steel fragment was 2.7 km/s. Under the threshold velocity condition, the two composite-structure reactive fragments increase the time and intensity of the compressed shock wave pulse in the explosive due to the impact energy release effect of the reactive materials, causing the shielded charge to detonate under the continuous long-term pulse loads. However, the mechanism of the steel fragment on the shielded charge belongs to the shock–detonation transition. The research results can provide scientific references for the design of hypervelocity reactive fragments and the study of their damage mechanism.

## 1. Introduction

Traditional tungsten alloy fragments or steel alloy fragments mainly rely on kinetic energy to penetrate and damage targets. This effect is singular, and sometimes, the target is hit but not destroyed. Due to the limitations of the materials’ own properties and damage modes, it is very difficult to further improve their damage power. In recent years, reactive materials have attracted the attention of various powerful countries, and the types of reactive materials have diversified, with examples including thermite, metal/polymer mixtures [1,2,3], intermetallic compounds [4,5], Zr-based amorphous alloys [6,7,8,9,10], and high-entropy alloys [11,12,13,14]. The damage elements made of these materials have the dual damage effect of kinetic energy penetration and chemical energy release. In addition, they are also functionally enabled to ignite and detonate flammable and explosive materials. The reactive material fragments act on the shielded charge, have an enhanced initiation effect, and can realize high-efficiency damage to the shielded charge.

In the field of terminal damage, reactive materials based on fluoropolymers and reactive metals (such as PTFE/Al, PTFE/Zr, and PTFE/Al/W) exhibit a significantly superior effect. Liu et al. [15] found that a projectile filled with Al/PTFE/W reactive materials caused serious rupture damage to multi-spaced aluminum plates behind the front steel target under the condition of matching the appropriate front target; the combined damage mechanisms of kinetic and chemical energy were the main cause of this damage. The experiments of Zhang et al. [16] demonstrated that PTFE/Al fragments have an obvious reaming effect on light targets at a speed of about 800 m/s due to the strong deflagration reaction. However, when the W element was added to PTFE/Al, the reaming ability of the fragments decreased with the increase in W content; the main reason for this concomitant decrease was that the addition of W weakened the chemical reaction of the reactive material. In the range of 1200–1500 m/s, the chemical energy ignition mechanism of the PTFE-based reactive material fragments is the main determinant of the shielded charge explosion, and as a result of the large amount of chemical energy generated by the impact, the reactive fragments are more likely to detonate the shielded charge compared to inert metal fragments [17]. In order to impact detonate a warhead, the inert fragments need to perforate the warhead shell and maintain a high residual velocity; however, the PTFE-based reactive fragment only needs to perforate the warhead shell, which releases a large amount of chemical energy into the explosive, providing an ignition mechanism similar to the igniter. Compared with a copper-shaped charge jet, the penetration depth of a PTFE/Al jet into armor is reduced, but the penetration aperture is increased. This reaming effect is mainly caused by the deflagration reaction of the PTFE/Al jet. This reveals that the PTFE/Al jet possesses a significant explosive damage effect [18,19]. When the PTFE/Zr long-rod penetrator strikes a thin target at hypervelocity, a reactive debris cloud forms behind it. The higher the impact velocity, the more severe the destruction that is caused by the reactive debris cloud on the multi-layer aftereffect targets [20]. This is because a higher impact velocity results in a more fully broken reactive material penetrator and more intense detonation-like reaction. Although Tang et al. [21] studied the visible light thermal radiation characteristics and plasma characteristic parameters of PTFE/Al fragments after hypervelocity impact on thin aluminum plates, the damage effect of the fragments on target plates has not been studied in detail. For the PTFE/Al Whipple shield, under the impact of a 5 km/s aluminum alloy projectile with a diameter of 6.4 mm, only the PTFE/Al in the high-pressure area was broken, and the detonation reaction occurred because the PTFE/Al cannot undergo a self-sustaining reaction. The shock wave generated by the detonation reaction of the PTFE/Al bumper can effectively decelerate and crush the projectile, causing the debris cloud to expand rapidly, increasing the impact area on the rear wall, reducing the speed of the center of mass of the projectile debris, and thereby reducing the damage caused by the debris cloud to the rear wall [22].

Currently, numerous research studies have focused on PTFE/Al jet penetration and the penetration of a PTFE/Al fragment and its composite-structure fragment within 1.6 km/s. In the field of hypervelocity collision, there are only a few studies on the use of the PTFE/Al material as a spacecraft bumper [22,23,24,25], but there is almost no research on fragments. The collision between PTFE/Al prefabricated fragments and a high-speed flying target, with a relative velocity that is more than 2 km/s, is classified as an ultra-high-speed collision [26]. When PTFE/Al fragments or PTFE/Al composite-structure fragments impact a shielded charge at hypervelocity, the energy release reaction, impact initiation mechanism, and initiation process of the shielded charge are not clear. Therefore, it is necessary to study the ultra-high-speed collision problems inherent in PTFE/Al composite-structure reactive fragments against a shielded charge.

In the current technical environment, it is neither economical nor realistic to conduct a large number of hypervelocity impact tests of fragments at 2 km/s or more, whereas numerical simulations can qualitatively study the hypervelocity impact process of fragments on targets. Hence, the experiment in reference [21] was simulated to verify the validity of the Lee–Tarver model of PTFE/Al material, the steel fragment was then used for a comparison, and the Lee–Tarver model was used to simulate the hypervelocity impact initiation process of two PTFE/Al composite-structure reactive fragments against a shielded charge. Through numerical simulation, the impact initiation mechanism and initiation process of two kinds of hypervelocity composite-structure reactive fragments on the shielded charge were revealed, the enhancement effect of the energy release reaction of the PTFE/Al in the process of ultra-high-speed impact initiation was analyzed, and the threshold velocity of the composite-structure reactive fragments impact initiation on the shielded charge was obtained.

## 2. Numerical Simulation Model and Reliability Verification

### 2.1. Simulation Model

**Figure 1:**
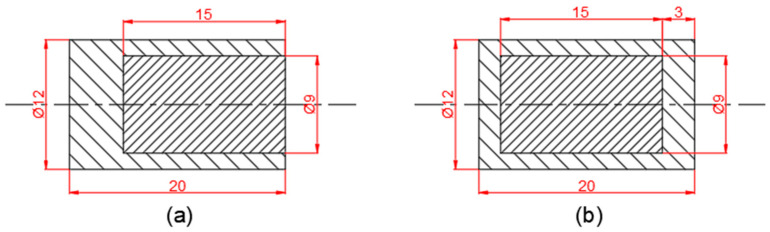
Schematics of composite-structure reactive fragments: (**a**) type I; (**b**) type II (unit: mm).

As shown in Figure 1, the two composite-structure reactive fragments were a steel-semi-coated PTFE/Al fragment (noted as type I) and a steel-coated PTFE/Al fragment (noted as type Ⅱ), which were cylindrical in a shape of φ1.2 cm × 2 cm and consisted of two parts of an outer casing and an inner reactive core. The outer casing material was 4340 steel, and the inner core material was PTFE/Al. The head of the type Ⅰ fragment had no shell, while the head of the type Ⅱ fragment had a coated shell. The shell of the shielded charge was 4340 steel with a thickness of 1.3 cm and an outer diameter of 37.2 cm. The charge was Comp B explosive with a diameter of 34.6 cm [27].

**Figure 2:**
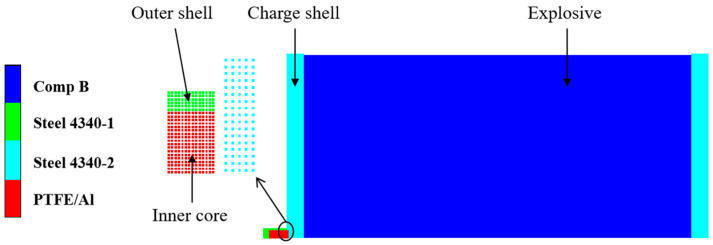
One-half simulation model of type I fragment vertically impacting shielded charge.

A two-dimensional axisymmetric simulation analysis was carried out using ANSYS/AUTODYN 17.0 software with the unit system of cm-g-μs. The SPH algorithm was adopted to model the fragments, shielded charge shells, and explosives. The one-half simulation model of the type I composite-structure reactive fragment vertically colliding with a shielded charge is shown in Figure 2. The initial boundary conditions of the shielded charge shells and explosive were set to “X-Velocity”, and their values were 0. (Under other simulation conditions, the definition of the initial boundary condition of the target was the same as this.)

### 2.2. Material Models and Parameters

The research has indicated that for hypervelocity impact problems, the equation of state (EOS) of the material has a crucial effect on the evolution of the debris cloud; however, the strength and failure models have no significant influence on it [22]. Thus, the failure model of the material was ignored in the simulation. The EOS of the PTFE/Al reactive material adopted the Lee–Tarver model, which was used to simulate the detonation reaction behavior of the reactive material under the hypervelocity impact condition. The model includes a trinomial reaction rate equation and JWL and Shock equations. Among them, the JWL equation was used to describe the reaction gas products, and the Shock equation was used to describe the unreacted PTFE/Al material.

The reaction rate equation can be expressed as

(1) $$\frac{\partial F}{\partial t}=I{\left(1-F\right)}^{b}{\left(\frac{\rho }{\rho_{0}}-1-a\right)}^{x}+G_{1}{\left(1-F\right)}^{c}F^{d}P^{y}+G_{2}{\left(1-F\right)}^{e}F^{g}P^{z}$$

The first item on the right side of the equation represents the formation of hot spots and the ignition of the heating zone in the reactive material. The second item represents the slow reaction process inward or outward after the formation of hot spots. The third item represents the rapid completion of the detonation reaction. In the formula, *F* is the reaction fraction, that is, the degree of reaction, which controls the release of chemical energy in the reactive material during the simulated detonation process. *F* = 0 indicates that no reaction occurs, and *F* = 1 indicates that a complete reaction has occurred. *t* is the time; *ρ*_0_ and *ρ* are the initial density and the current density, respectively; *P* is the pressure; and *I*, *b*, *a*, *x*, *G*_1_, *c*, *d*, *y*, *G*_2_, *e*, *g*, and *z* are constants related to the material. Among these parameters, *a* is the critical compressibility used to prevent ignition; *I* and *x* control the amount of ignition, which is a function of impact strength and duration; *G*_1_ and *d* control the early growth of the reaction after ignition; and *G*_2_ and *z* determine the high-pressure reaction rate. Since PTFE/Al does not have a self-sustaining reaction ability, *G*_2_ = 0 in this model.

The JWL equation is

(2) $$P=A(1-\frac{\omega }{R_{1}V})e^{-R_{1}V}+B(1-\frac{\omega }{R_{2}V})e^{-R_{2}V}+\frac{\omega E}{V}$$

where *P* is the pressure of detonation products; *V* is the relative specific volume; *E* is the specific internal energy per unit mass; and *A*, *B*, *R*_1_, *R*_2_, and *ω* are the material parameters.

The Shock equation is

(3) $$D_{S}=c_{0}+\lambda u_{P}$$

where *D_S_* is the propagation velocity of the shock wave in the material; *c*_0_ is the volume sound velocity of the material; *u_P_* is the particle velocity in the material; and *λ* is the material coefficient.

The Johnson–Cook equation was chosen for the strength model of PTFE/Al. The mass ratio of PTFE to aluminum in PTFE/Al was 73.5/26.5, and the average particle sizes were 3 μm and 30 μm, respectively. The content and particle size of the components of PTFE/Al used in this study were the same as those in the literature [22,23], so the EOS and strength model parameters of the PTFE/Al material could be selected from the literature; they are shown in Table 1 and Table 2, respectively [22].

The EOS of 4340 steel used the Shock equation, the strength model used the Johnson–Cook equation, and the material parameters were derived from references [27,28], as shown in Table 3. The EOS of the Comp B explosive adopted the Lee–Tarver model, and the strength model adopted the von Mises model. The parameters were taken from the material library of AUTODYN.

### 2.3. Reliability Verification of Material Model

**Figure 3:**
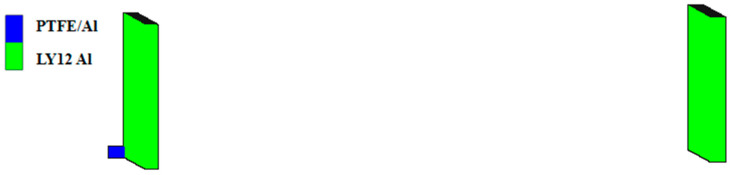
Quarter simulation model of the validation experiment.

To verify the reliability of the PTFE/Al material model, the experiment described in the literature in reference [21] was simulated and analyzed using the SPH algorithm. The size of the PTFE/Al cylindrical fragment was Φ 0.5 cm × 0.5 cm, the thickness of the front LY12 aluminum target was 0.3 cm, the thickness of the rear LY12 aluminum target was 0.4 cm, the length and width of the two targets were 10 cm, and the distance between the two targets was 20 cm. The fragment impacted the aluminum target at a speed of 2.23 km/s, and a three-dimensional quarter simulation model is shown in Figure 3. The initial boundary condition of the two aluminum targets was set to “X-Velocity”, and the value was 0. In the simulation, the material models and parameters mentioned previously were used for PTFE/Al, and the Tillotson equation of the state and the Johnson–Cook strength model were used for LY12 aluminum, with the material parameters obtained from reference [29].

**Figure 4:**
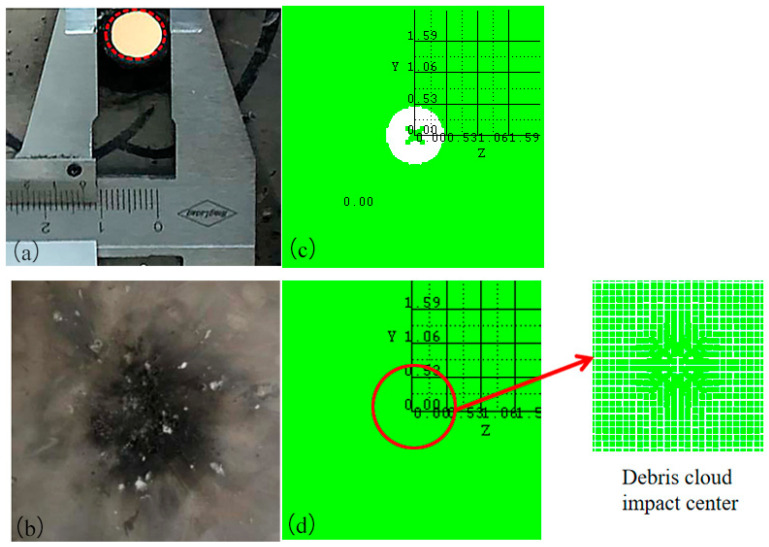
Damage results of experiment [21] and simulation target plates: (**a**) the perforated front target in experiment; (**b**) rear target with small craters and black smoke in experiment; (**c**) the perforated front target in simulation; (**d**) debris cloud impact center on the rear target in simulation.

The size of the SPH particles has a significant influence on the simulation results. If the particles are too large, the error in the calculation results is large; on the contrary, too small particles will increase the calculation time. After several trial calculations, the particle diameters of the fragment and the target plates were determined to be 0.25 mm and 0.50 mm, respectively, which permits both the calculation accuracy and time to be taken into account. The damage results of the experimental and simulated target plates are shown in Figure 4.

As shown in Figure 4a,b [21], in the experiment, a detonation reaction occurs when the PTFE/Al reactive fragment impacts the front target at hypervelocity. Under the combined action of kinetic energy and chemical energy, the fragment perforates the front target and produces a reaming effect, with a perforation diameter of 1.2 cm. The debris cloud formed by the hypervelocity impact on the front target does not perforate the rear target, and produces only some small pits and a large amount of black smoke on it. As shown in Figure 4c,d, the perforation diameter of the front target obtained through numerical simulation was 1.06 cm. Compared with the experimental results, the error was −11.67%, and the absolute value was less than 15%. The rear target was not perforated; there was only slight damage to the central area of the debris cloud impact, which tends to be negligible. From the damage to the front and the rear target, the simulation results are in good agreement with the experimental results. This demonstrates that the Lee–Tarver model of PTFE/Al has certain reliability and can be used for the subsequent numerical simulation research.

**Figure 5:**
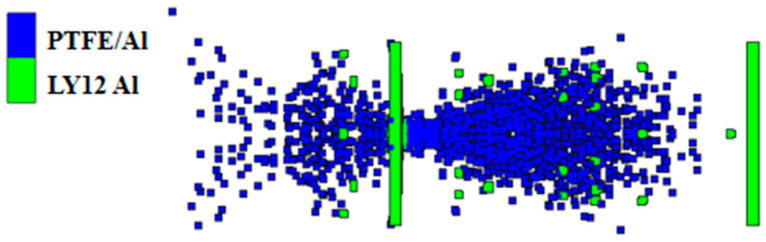
Distribution of debris cloud at *t* = 100 μs.

**Figure 6:**
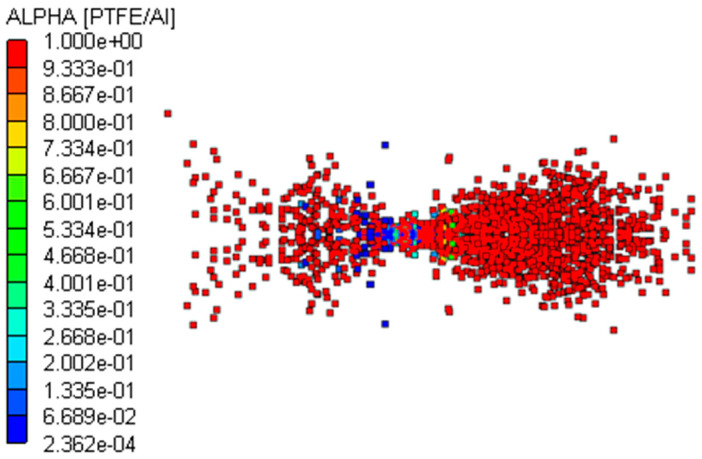
Reactivity contour diagram of PTFE/Al at *t* = 100 μs.

When the PTFE/Al reactive fragment impacted the front target at hypervelocity, a high pressure was generated on the contact surface that propagated rapidly backward in the form of a shock wave. The pressure in the fragment was higher than the reaction threshold pressure of PTFE/Al, and the reactive fragment was activated to undergo a detonation reaction while penetrating the front target. After penetrating the front target, it formed a reaction debris cloud. When the debris cloud moved to the rear target, it had reacted completely to form detonation products. Some of the carbons in the detonation products were attached to the rear target; that is, there was a large amount of black smoke on the rear target, so the reaction debris cloud could not damage the rear target. When the reactive fragment collided at hypervelocity and penetrated the front target, the front target material formed some high-speed fragments. These fragments were larger than the reaction debris, so they demonstrated only a slight penetration into the rear target and formed some small pits. As shown in Figure 5 and Figure 6, the formation mechanism of the black smoke and small pits on the rear target could be adequately explained using numerical simulations, which once again proved the reliability of the PTFE/Al material model.

### 2.4. Convergence Analysis and Verification

According to the analysis in Section 2.3, when simulating the hypervelocity impact initiation of the shielded charge by the composite-structure reactive fragments, the SPH particle sizes of the fragment and the charge shell should be set to 0.25 mm and 0.50 mm, respectively. To improve the accuracy of the simulation calculation, it is necessary to analyze the convergence of the charge particle size. In the convergence analysis, the fragment material was PTFE/Al, the size was the same as that of the type I fragment in Section 2.1, and the shielded charge model was unchanged. In the simulation, the PTFE/Al material was considered to be inert, and the Shock equation of the state and the Johnson–Cook strength model were used. The purpose of this treatment was to facilitate the theoretical verification of the convergence of the particle size of the charge.

#### 2.4.1. Theoretical Analysis

**Figure 7:**
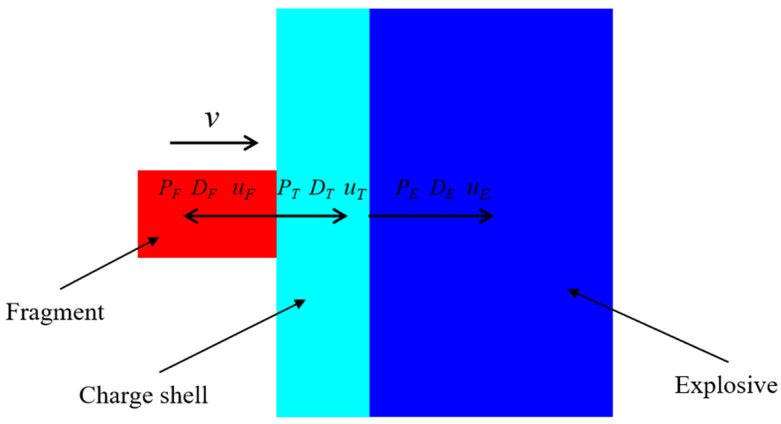
Simplified analysis model of the PTFE/Al fragment hypervelocity impact on the shielded charge.

The PTFE/Al fragment vertically impacted the shielded charge at a velocity of *v* = 3 km/s. As shown in Figure 7, two shock waves were generated at the interface at the moment of impact, that is, the left and the right shock waves propagating from the interface to the fragment and the shielded charge shell, respectively. According to the law of conservation of momentum,

(4) $$P_{T}=\rho_{T}D_{T}u_{T}$$

(5) $$P_{F}=\rho_{F}D_{F}u_{F}$$

where *P* is the pressure of the initial shock wave produced by the collision; *D* is the velocity of the shock wave; *u* is the particle velocity in the compression zone; *ρ* represents the material density, and subscripts *F* and *T* refer to the fragment and the charge shell, respectively. From the interfacial continuity conditions, it can be obtained that

(6) $$P_{T}=P_{F}$$

(7) $$u_{T}+u_{F}=v$$

The shock wave velocity and the particle velocity satisfy the following relationships:

(8) $$D_{F}=c_{F}+\lambda_{F}u_{F}$$

(9) $$D_{T}=c_{T}+\lambda_{T}u_{T}$$

where *c* represents the volume sound velocity, and *λ* is the material coefficient. In combining the above six equations, *u_T_* (0 < *u_T_* ≤ *v*) can be obtained, and then *P_T_*. The parameters required for calculation are shown in Table 1 and Table 3.

When the initial right shock wave generated by the collision propagates to the interface between the shielded charge shell and the explosive, the pressure attenuates to *P_T_*_1_, and the attenuation law is represented as follows [30]:

(10) $$P_{T1}=P_{T}\exp(-\alpha h)$$

where *α* is the shock wave pressure attenuation coefficient of the charge shell material with a value of 0.0536/mm [31], and *h* is the distance of the shock wave motion, which is the wall thickness of the charge shell. At the interface, the shock wave pressure *P_T_*_1_ and the particle velocity *u_T_*_1_ in the charge shell satisfy the following relationship:

(11) $$P_{T1}=\rho_{T}(c_{T}+\lambda_{T}u_{T1})u_{T1}$$

The shock wave enters the explosive from the shell and forms a transmitted shock wave in the explosive. The transmitted shock wave pressure *P_E_* is

(12) $$P_{E}=\rho_{E}(c_{E}+\lambda_{E}u_{E})u_{E}$$

where *u_E_* is the particle velocity of the explosive at the interface, and *ρ_E_*, *c_E_*, and *λ_E_* are the density, volume sound velocity, and material coefficient of the explosive, respectively, with values of 1.717 g/cm^3^, 2.71 km/s, and 1.86 [32]. In accordance with the interfacial continuity conditions of the reflection and transmission of the shock wave at the interface of two media with different impedances, it can be seen that

(13) $$P_{E}=\rho_{T}[c_{T}+\lambda_{T}(2u_{T1}-u_{E})](2u_{T1}-u_{E})$$

The *u_T_*_1_ can be obtained using Equations (10) and (11). Through a combination of Equations (12) and (13) and the substitution of *u_T_*_1_ into them, *u_E_* can be solved. Then, the transmission shock wave pressure *P_E_* can be calculated. When *v* = 3 km/s, the corresponding *P_E_* value is 4.63 GPa (0.0463 Mbar).

#### 2.4.2. Convergence Analysis

**Figure 8:**
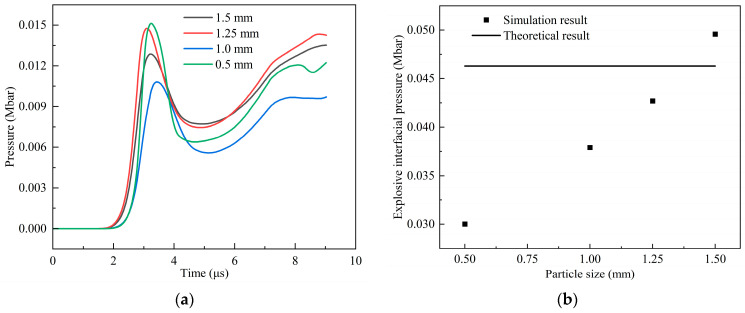
Pressure history curves and pressure values of different particle sizes: (**a**) pressure history curves of point A; (**b**) pressure values of point B.

**Figure 9:**
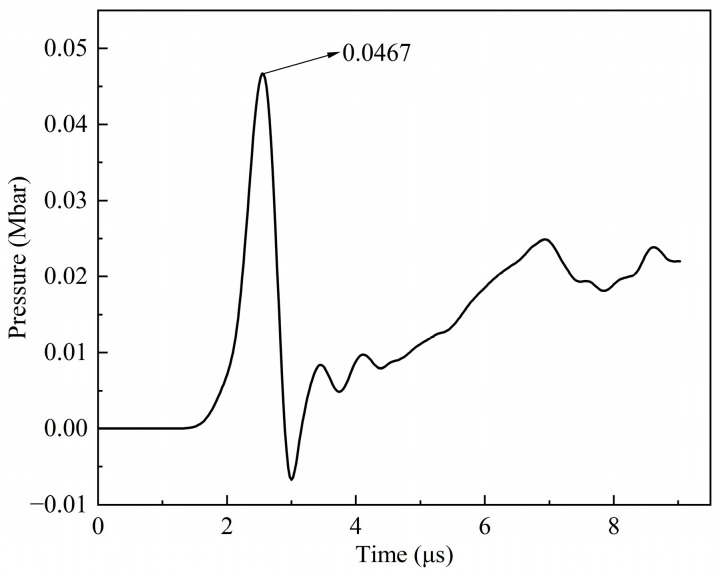
Pressure history curve of point B when the explosive particle size is 1.4 mm.

In order to obtain the pressure values at specific positions in the charge, point A and point B were set on the symmetry axis. Point A was in the explosive, 0.2 cm away from the interface between the shell and the explosive, and point B was located on this interface. Firstly, the particle sizes of the charge were set to 0.5 mm, 1.0 mm, 1.25 mm, and 1.5 mm for the simulation analysis. Figure 8a shows the pressure history curves of point A for different particle sizes when the PTFE/Al fragment vertically impacts the shielded charge at a speed of 3 km/s. On the whole, the results achieved using 1.25 mm and 1.5 mm particle sizes are close to each other. Combined with Figure 8b, it can be clearly seen that the theoretical pressure value of point B is between the simulation results of 1.25 mm and 1.5 mm. Compared with the theoretical value of point B, the errors in these two simulation results were −7.78% and 7.11%, respectively. Therefore, the particle size of 1.4 mm is again selected for the simulation verification. Figure 9 shows the pressure history curve of point B when the particle size is 1.4 mm. When the shock wave generated by the collision moves to point B, the pressure is 0.0467 Mbar, and the error is 0.86%, compared with the theoretical value. Therefore, the size of the explosive particles in the subsequent simulations was set to 1.4 mm.

The pressure of point B obtained by setting the explosive particle size to 1.4 mm was almost the theoretical value. The reason is that if the particle size is too small, it cannot provide enough force to the particle in the search domain, and thus, it reduces the calculation accuracy; if its value is too large, the detailed and local characteristics of the particle may be flattened, which will also reduce the accuracy. So, it is necessary to select the appropriate particle size. This is different from the Lagrange algorithm in which the calculation accuracy decreases with an increase in the element size.

## 3. Results and Analysis

When simulating and analyzing the hypervelocity impact shielded charge of the composite-structure reactive fragments and the steel fragment, a set of observation points were set at equal intervals on the horizontal symmetry axis of the explosive, with an interval distance of 1.5 cm. The first observation point was located on the interface between the shell and the explosive. They were numbered 1, 2, 3…, from the interface to the inside of the explosive.

### 3.1. Impact of Type I Fragment

**Figure 10:**
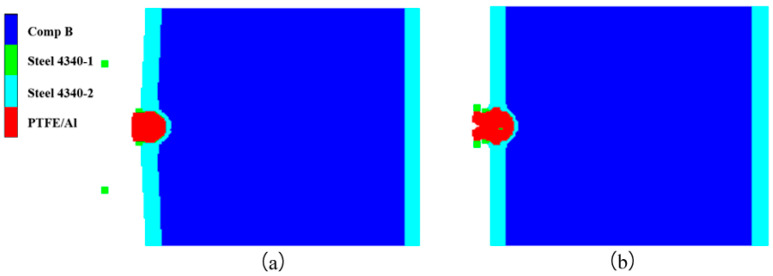
Deformation of type I fragment and shielded charge at *t* = 30 μs: (**a**) *V_P_* = 3.0 km/s; (**b**) *V_P_* = 2.3 km/s.

**Figure 11:**
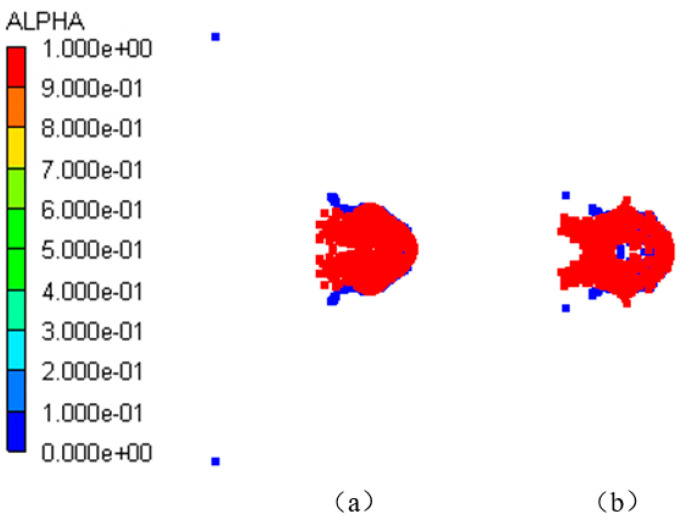
Reactivity contour diagram of the reactive core of type I fragment at *t* = 30 μs: (**a**) *V_P_* = 3.0 km/s; (**b**) *V_P_* = 2.3 km/s.

Continuous simulations of different impact velocities were performed to determine the impact initiation threshold velocity of the type I composite-structure reactive fragment on the shielded charge. First, the dynamic responses of the charge shell and explosive were studied at the two impact velocities of *V_P_* = 3.0 km/s and *V_P_* = 2.3 km/s. Figure 10 shows the deformation of the fragment, charge shell, and explosive at *t* = 30 μs. Obviously, the fragment penetrates the shielded charge at two impact velocities; however, the charge shell does not form a through hole, and it bulges only near the explosive side. The fragment shell is gradually worn and consumed during the penetration process, and the reactive core undergoes a detonation reaction. Figure 11 provides a reactivity contour diagram of the reactive core. The explosive may explode under the impact of a 3.0 km/s fragment. The expansion of the explosive and the deflection of the shell indicate this speculative result. Meanwhile, the explosive does not expand under the impact of 2.3 km/s, which indicates that the explosive failed to detonate in this case. It can be inferred that the impact initiation threshold velocity of the type I fragment on the shielded charge is between 2.3 km/s and 3.0 km/s.

**Figure 12:**
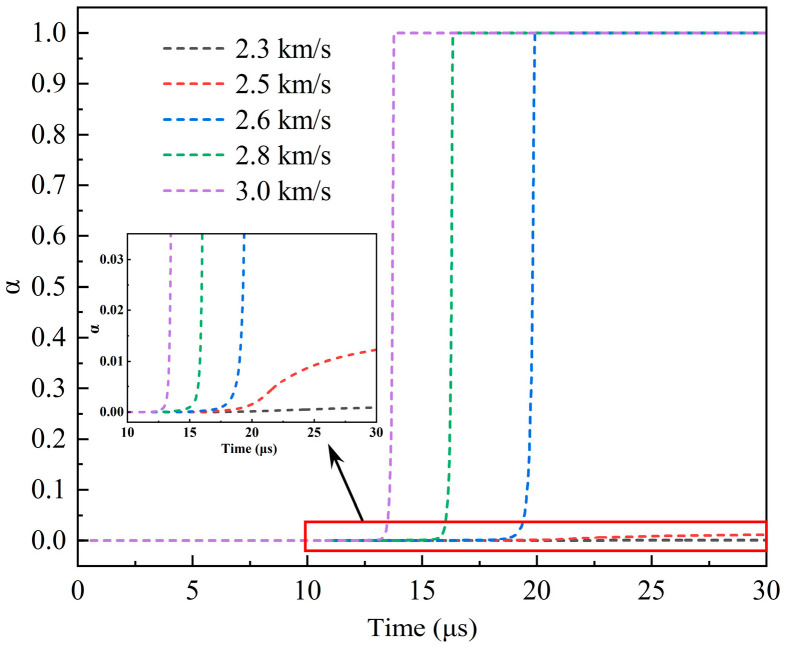
Reactivity history curves of observation point 3 at different impact velocities.

In addition to 2.3 km/s and 3.0 km/s, the impact velocities of 2.5 km/s, 2.6 km/s, and 2.8 km/s were simulated. The reactivity history curves of the third observation point in the explosive at five impact velocities are shown in Figure 12. *α* = 1 indicates that the reaction fraction of the explosive reaches 100%, and *α* = 0 indicates that there is no reaction. It can be seen from the figure that when the impact velocity of the type I fragment reaches 2.6 km/s, the explosive reactivity is 1. and the time of its change from 0 to 1 is very short (less than 5 μs). This demonstrates that the explosive reacts rapidly at 2.6 km/s. At velocities greater than 2.6 km/s, the initiation time of the explosive reaction gradually advances with an increase in the impact velocity, which is mainly due to the higher speed of the collision increasing the intensity of the shock wave in the shielded charge. At 2.5 km/s, the reaction fraction of the explosive is very small, only 1.2%, while at 2.3 km/s, the reaction fraction of the explosive is almost zero. Therefore, it is certain that these two speeds cannot cause the explosive to detonate. Through the analysis of explosive reactivity, it can be seen that the impact initiation threshold velocity of the type I fragment on the shielded charge may be 2.6 km/s.

**Figure 13:**
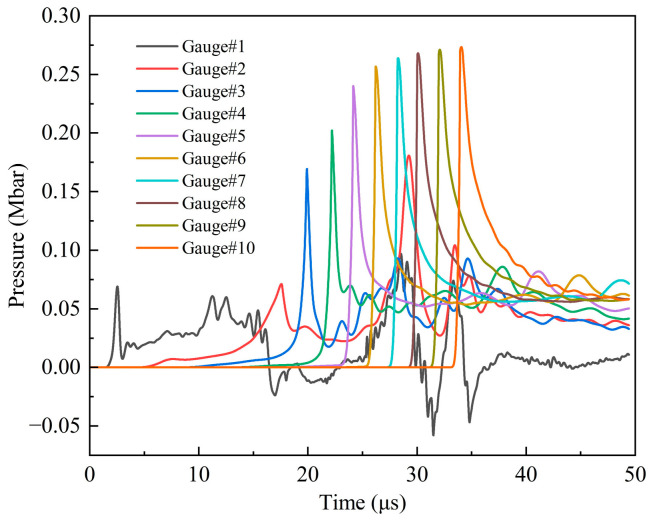
Pressure history curves of observation points 1–10 under impact of type I fragment at 2.6 km/s.

**Figure 14:**
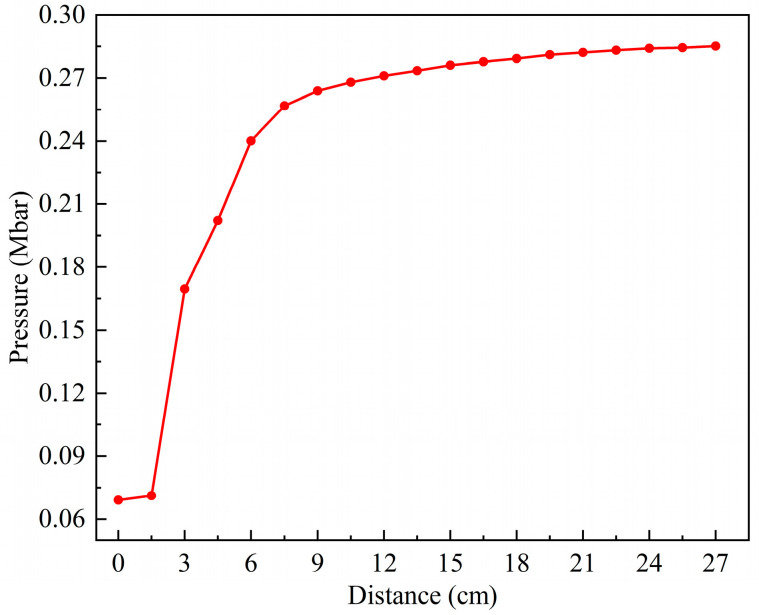
The first peak pressure curve at different positions under the impact of type I fragment at 2.6 km/s.

**Figure 15:**
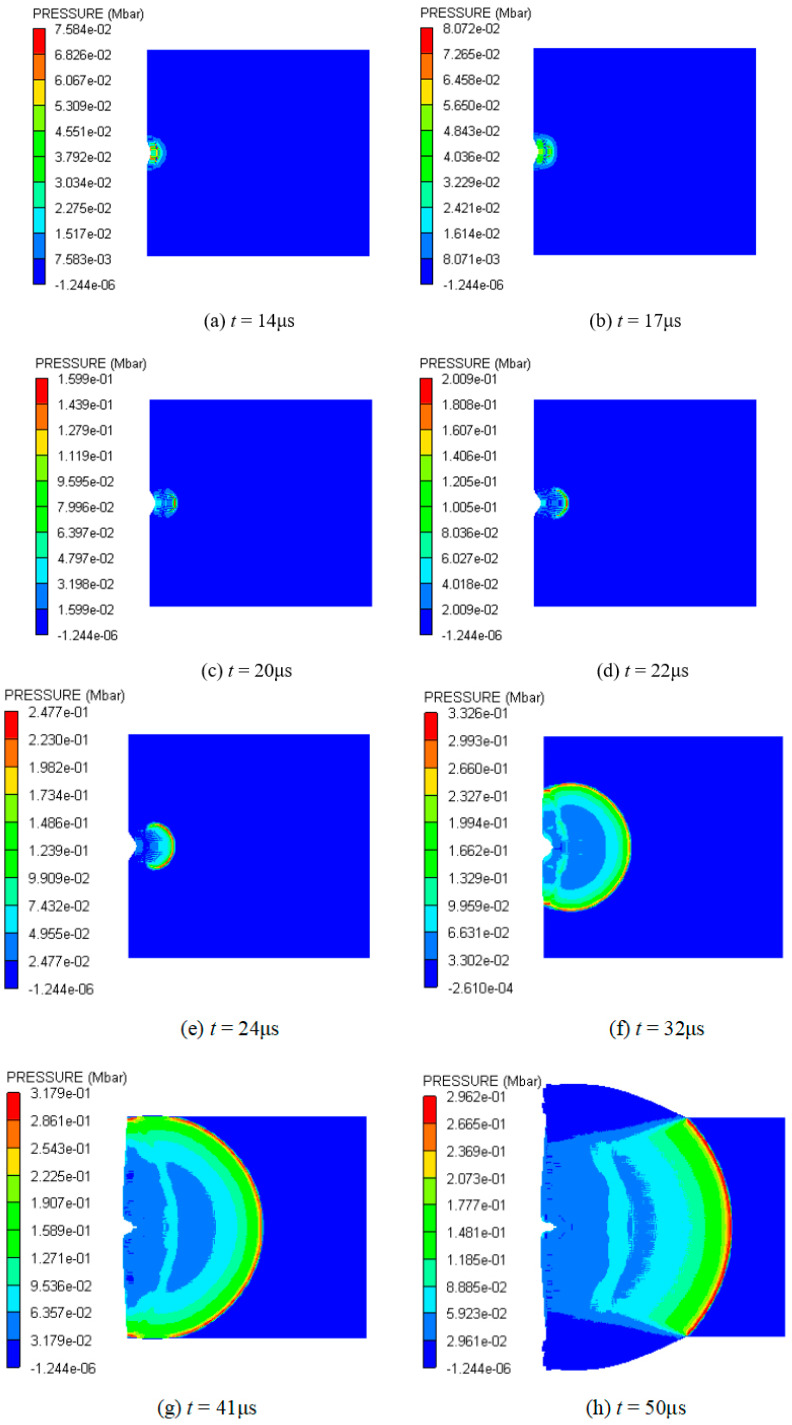
Pressure contour diagrams of explosive under the impact of type I fragment at 2.6 km/s.

Figure 13 shows the pressure history curves of 1–10 observation points in the explosive at 2.6 km/s. Significantly, from the third observation point, the first peak of each curve is the maximum pressure of each point, and then it gradually decreases from the peak. With the increase in the distance between the observation point and the shell–explosive boundary, the maximum pressure first increases rapidly, and then gradually tends to become gentler. The maximum pressure of observation point 9 is 0.271 Mbar, and the error is −8.1% compared with the C–J pressure of the Comp B explosive (0.295 Mbar), and its absolute value is within 10%, so the simulation results are reliable. The reason for the smaller simulation value is that there are some defects in the SPH algorithm itself. When the shock wave passes through the interface of two media with a significant density difference, the pressure will have a slight anomaly. In excluding the defects of the algorithm itself, it can be considered that the explosive begins to be in a stable detonation state at observation point 9. There is always a process from the external stimulation to the formation of a stable detonation wave, which is related to the initial external conditions and the characteristic detonation velocity of the explosive [33]. The shock wave formed through the impact of the fragment on the shell of the shielded charge propagates into the explosive. The velocity of the shock wave is less than the characteristic detonation velocity of the Comp B explosive and greater than its critical detonation velocity; therefore, there is an unstable detonation zone in the explosive. Since the shock wave velocity is greater than the critical detonation velocity of Comp B, the detonation of the explosive in the unstable detonation zone can continue and gradually strengthen and eventually develop into a stable detonation. This is the fundamental reason why the pressure peak gradually increases from observation point 3 to 9 and then remains basically unchanged. It can be seen that observation points 3–9 form an unstable detonation zone, and after, point 9 becomes a stable detonation zone. Figure 14 illustrates the first peak pressure curve at different positions in the explosive at 2.6 km/s. The horizontal axis represents the distance from the shell–explosive interface, and the abscissa of the data points on the curve corresponds to the positions of the observation points. It is worth noting that for observation point 2, the peak pressure of the shock wave generated by the collision is very small, so it is neglected. The obvious peak that follows it is regarded as the first peak pressure, and the ensuing analysis also adopts this consideration. From the diagram, it can be clearly seen that the pressure increase rate in the unstable detonation zone changes from fast to slow, which indicates that the growth rate of detonation in this zone changes from fast to slow. The peak pressure curve begins to flatten from 12 cm (point 9) away from the interface, indicating that the detonation has developed into a stable detonation, that is, a C–J detonation. The first peaks of observation points 1 and 2 in Figure 13 do not represent their maximum pressure, and the first peak pressures of these two points are quite different from the maximum pressure of the subsequent observation points, which is similar to the sudden change, as shown in Figure 14. Consequently, it can be considered that although the explosive at these two points reacts completely, no detonation is formed. The detonation starts from observation point 3, as shown in the pressure contour diagrams of the explosive in Figure 15, which prove this conclusion (see below for a detailed discussion). The maximum pressure values of observation points 1 and 2 in 25–30 μs are the high pressures generated through the interaction of the detonation products at these two points with the shielded charge shell under the driving force of the subsequent detonation product expansion.

Figure 15 shows a pressure nephogram of the explosive based on only the cancellation of other materials at 2.6 km/s. From 14 μs to 17 μs, the shock wave in the explosive propagates only in the horizontal direction, and its width basically does not change, as shown in Figure 15a,b. At 20 μs, it further propagates into the interior of the explosive, and the head becomes thicker and has a spherical waveform, as shown in Figure 15c. At this time, the shock wave front has moved into the vicinity of observation point 3. At 22 μs and 24 μs, the front of the shock wave becomes close to observation points 4 and 5, respectively. From Figure 15d,e, it can be seen that the spherical shock wave becomes larger rapidly and its pressure increases faster. The formation and evolution of the spherical shock wave in the explosive indicate that detonation begins at observation point 3. The explosive begins to be in a stable detonation at about 32 μs. At this time, the front of the spherical detonation wave acts on the shielded charge shell, and the pressure of the action zone is about 0.333 Mbar; meanwhile, the pressure of other positions in the detonation wave front is C–J pressure, as shown in Figure 15f. After that, the detonation wave shows a stable propagation, reaching the charge boundary on both sides at 41 μs, and rarefaction waves begin to enter from both sides, as shown in Figure 15g. However, the rarefaction waves do not affect the forward motion of the detonation wave in the horizontal direction, and the detonation remains stable, as shown in Figure 15h.

In analyzing the reactivity of the explosive, the first peak pressure, and the propagation of the detonation wave, it can be determined that the shielded charge can be successfully detonated under the impact of the 2.6 km/s type I fragment, and the explosion can be maintained until the explosive is consumed. This indicates that the threshold velocity of type I fragment impact initiation of the shielded charge is 2.6 km/s, and it also proves the correctness of the above approximate speculation of the threshold velocity range of impact initiation through the appearance deformation.

### 3.2. Impact of Type II Fragment

Based on the research results of the type I fragment, the impact initiation performance of the type II fragment at 2.6 km/s was simulated first; then, the subsequent simulations were carried out according to the following method: if the shielded charge is not detonated at 2.6 km/s, the impact velocity is increased; otherwise, the impact velocity is reduced.

**Figure 16:**
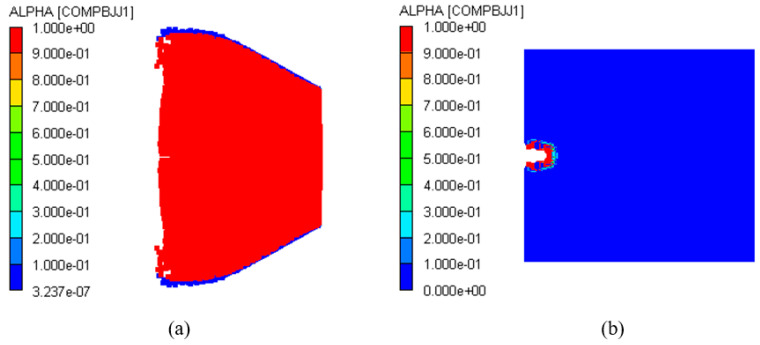
Reactivity contour diagrams of explosive under the impact of type II fragment: (**a**) *V_P_* = 2.6 km/s; (**b**) *V_P_* = 2.5 km/s.

The shielded charge was detonated with the type II fragment at 2.6 km/s; its reactivity contour diagram is shown in Figure 16a. So, the fragment velocity was reduced to 2.5 km/s for simulation. In this case, the reactivity contour diagram of the explosive at the end of the fragment penetration is shown in Figure 16b. It is obvious that the fragment perforates the shielded charge shell and enters into the explosive, but the explosive is not detonated. Therefore, 2.6 km/s is the threshold velocity of the type II fragment impact initiation of the shielded charge.

**Figure 17:**
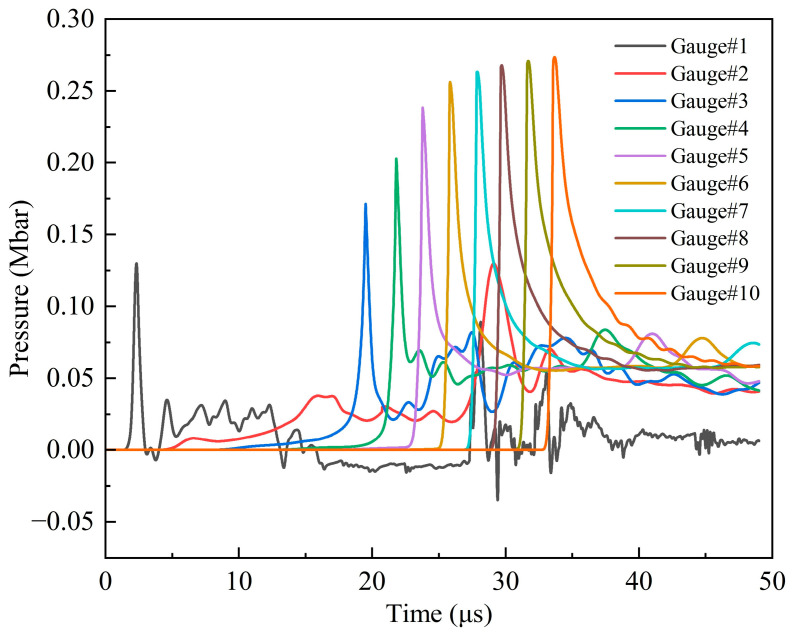
Pressure history curves of observation points 1–10 under the impact of type II fragment at 2.6 km/s.

Through the study of the type I fragment impact initiation of the shielded charge, it can be found that the impact initiation process can be reliably analyzed through the use of the pressure history curves of the explosive observation points. Therefore, the pressure history curves were selected for analysis, as shown in Figure 17. The curves show that the peak pressure increases rapidly at first and then tends to become gentler gradually from observation point 3, and the peak pressure at observation points 9 and 10 is essentially equal. This change phenomenon is consistent with the change in the pressure of the observation point in the explosive under the impact of the 2.6 km/s type I fragment, and the peak pressure at the same point is almost equal. So, it can be determined that the pressure at observation point 9 is the C–J detonation pressure of Comp B, and the explosive is stably detonated after that. From observation points 1 to 2, the first pressure peak decreases rapidly. This is because when the shock wave formed by the hypervelocity impact reaches the explosive interface, its peak value is still significant; however, the impedance of the explosive is smaller than that of the shell, so the shock wave propagates and attenuates very quickly in the explosive, and its value is very small at observation point 2. Under the penetration and extrusion of the fragment, the explosive can still react, and the pressure begins to rise. However, no detonation is formed, so the pressure is significantly smaller than that at observation point 1. From observation points 2 to 3, the first peak pressure increases rapidly, and the difference is large. The reason for this situation is that the explosive starts to detonate near point 3. Therefore, points 3–9 represent an unstable detonation zone, and the detonation of an explosive in this area is gradually strengthened. Through the analysis of the peak pressure, it can be seen that the threshold velocity of the type II fragment impact initiation of the shielded charge is also 2.6 km/s.

### 3.3. Impact of Steel Fragment

As a comparison, the length of the 4340-steel fragment was 1.39 cm, and its mass and diameter were exactly the same as those of the composite-structure reactive fragment. The method in Section 3.2 was used for the simulation.

**Figure 18:**
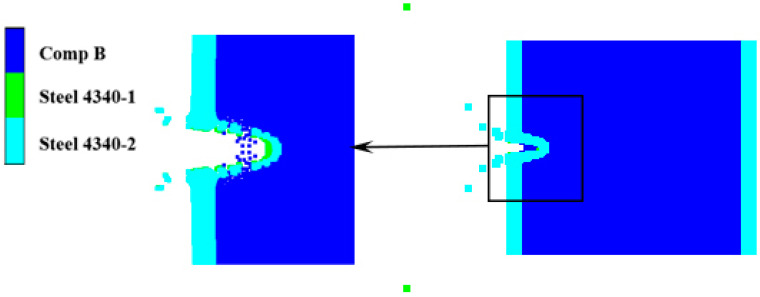
Penetration results of steel fragments at 80 μs.

**Figure 19:**
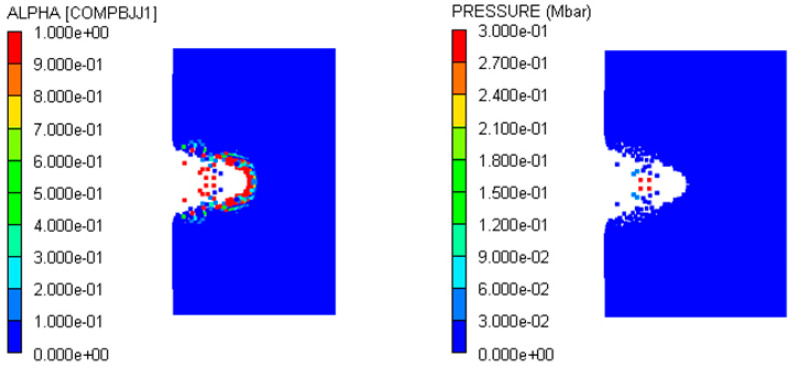
Local reactivity contour diagram and pressure contour diagram of explosive under the impact of steel fragment at 2.6 km/s (80 μs).

As shown in Figure 18, at 80 μs, the steel fragment with 2.6 km/s had perforated the shielded charge shell and entered the explosive. At this time, the velocity of both the residual fragment and the plug is zero. Under the impact and penetration of the steel fragment, the explosive does not expand, and the shell does not produce any obvious flexural deformation. Combined with the reactivity contour diagram and the pressure contour diagram of the explosive in Figure 19, it can be clearly determined that the shielded charge is not detonated at this impact velocity, although the steel fragment penetrates the shell and drills into the interior of the explosive.

**Figure 20:**
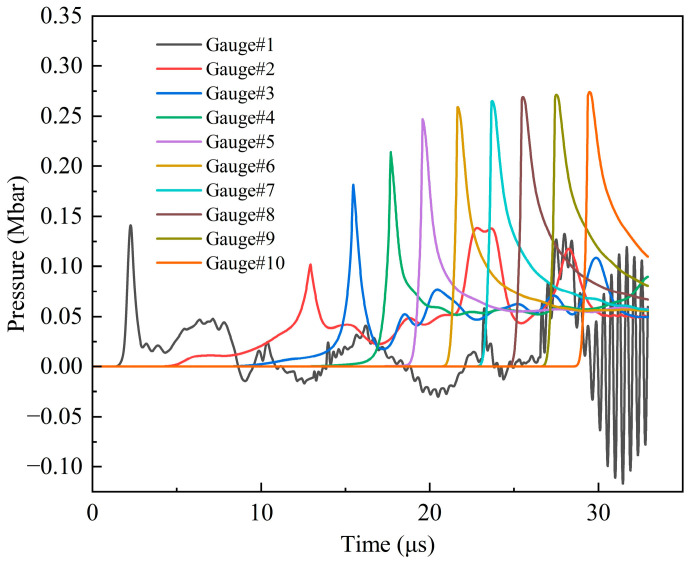
Pressure history curves of observation points 1–10 under the impact of steel fragment at 2.7 km/s.

**Figure 21:**
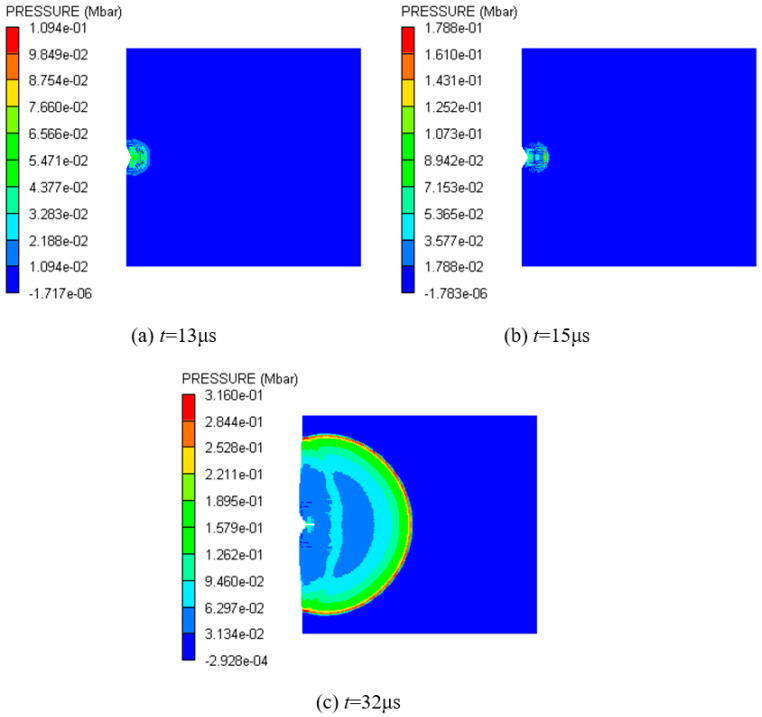
Pressure contour diagrams of explosive under the impact of steel fragment at 2.7 km/s.

The impact velocity of the steel fragment was increased to 2.65 km/s and 2.7 km/s for the simulations. Similarly, the 2.65 km/s steel fragment also failed to impact detonate the shielded charge, while the 2.7 km/s steel fragment was successful. Figure 20 shows the pressure history curves of observation points 1–10 at 2.7 km/s. With the exclusion of the first peak pressure values of observation points 1 and 2 and the time corresponding to the first peak value of observation points 2–10, the pressure history curves are similar to those of the type I and type II fragments on the whole. Compared with the impact of the two composite-structure fragments, because the fragment material is steel and the impact speed is high, the explosive reaction time at observation point 2 is advanced, and the complete reaction speed is faster, resulting in higher pressure. As a result, from observation points 2 to 3, the time for the explosive to form a detonation from a fast reaction is also advanced by about 4 μs. It is believed that the detonation of the explosive begins at observation point 3. The reason is that from observation points 2 to 3, the shock wave head changes significantly, and the whole contour image changes from a column to a mushroom shape; that is, the head forms a typical spherical waveform, such as that shown in Figure 21a,b. Similarly, observation points 3–9 represent the unstable detonation zone, and after observation point 9 is the stable detonation zone. Based on the above analysis, it can be concluded that 2.7 km/s is the threshold velocity of steel fragment impact initiation on the shielded charge. As shown in Figure 21c, under this impact velocity, the explosive can finally maintain stable detonation.

### 3.4. Analysis of Impact Initiation Mechanism of Three Fragments

**Figure 22:**
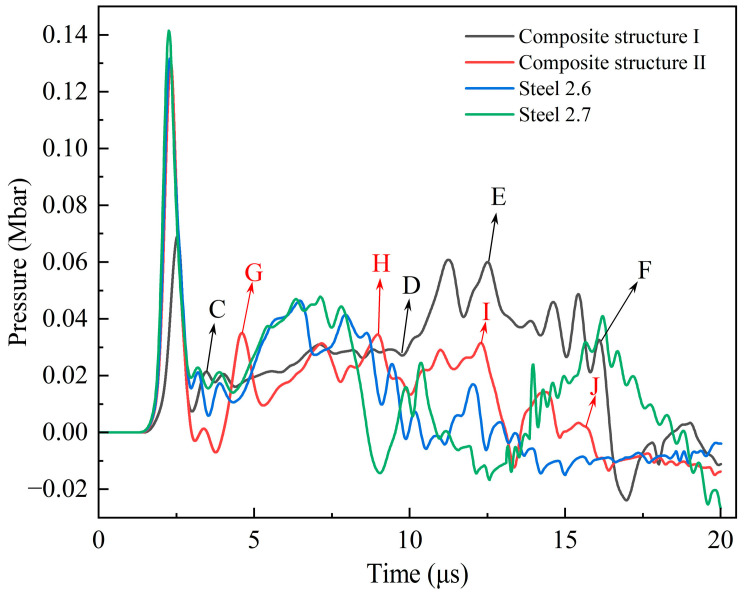
Pressure pulse at explosive interface.

**Figure 23:**
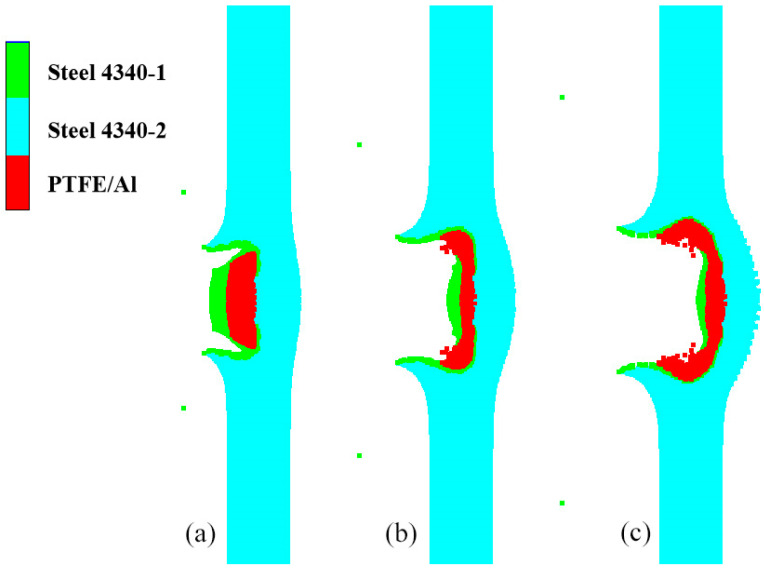
Penetration of type Ⅰ fragment into the shell of shielded charge: (**a**) 7 μs, CD segment; (**b**) 11 μs, DE segment; (**c**) 15 μs, EF segment.

**Figure 24:**
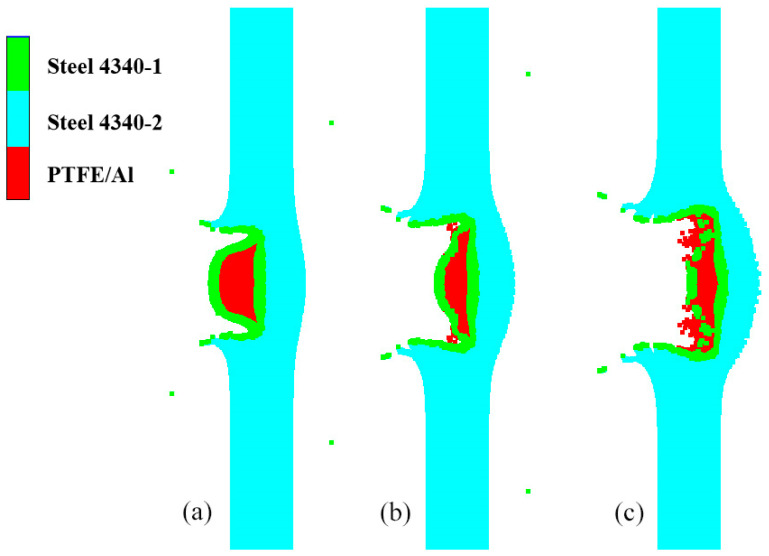
Penetration of type II fragment into the shell of shielded charge: (**a**) 7 μs, GH segment; (**b**) 11 μs, HI segment; (**c**) 15 μs, IJ segment.

For two kinds of composite-structure fragments with 2.6 km/s and steel fragments with 2.7 km/s impact the shielded charge shell, the pressure pulses generated at the explosive interface are shown in Figure 22. It can be seen from the collision dynamics that the larger the density of the fragment material and the Hugoniot parameter, the higher the initial collision pressure, so the pressure of the compression shock wave moving to the explosive interface is also higher. The part of the collision between the type II fragment and the shielded charge shell is steel. At the same impact velocity, the pressure that transmits to the explosive interface at the moment of collision is almost the same as that of the steel fragment, but larger than that of the type I fragment. According to the analysis in Section 3.3, it can be seen that the 2.6 km/s steel fragment cannot detonate the shielded charge, so for the two kinds of composite-structure reactive fragments, if the impact energy release effect is not considered, it is impossible to detonate the shielded charge using only kinetic energy. Obviously, the detonation reaction of the PTFE/Al material in the two composite-structure fragments during hypervelocity impact has a certain enhancement effect on the impact initiation. The impact initiation enhancement mechanisms of the two composite-structure fragments are as follows:

(1) The head of the type I fragment is not covered with the steel shell. During hypervelocity impact, the reactive material is broken, and the detonation reaction is excited. During the penetration process, the pressure generated through the expansion of the detonation products of the reactive material propagates through the charge shell to the explosive. At the beginning of penetration, the reactive material becomes excited and reacts rapidly. However, because the detonation products of the reactive material are in the initial stage of expansion, the pressure acting on the charge shell is very small, and the compressive shock wave transmitted into the explosive is mainly driven by the kinetic energy of the fragment. This is the CD segment of the curve in Figure 22, so the pressure of the shock wave is relatively low. With the further expansion of the detonation products and the destruction of the shell, the intensity of the compressed shock wave pulse imparted to the explosive increases and maintains a short-term stability, which is described with the DE segment of the curve in Figure 22. When the detonation products continue to expand inside the penetration hole and a large number of detonation products diffuse in the opposite direction, the shock wave intensity gradually decreases, such as in the EF segment. The partially enlarged pictures of the penetration process of the type I fragment at typical moments corresponding to these three stages are shown in Figure 23. After 10 μs, the explosive interface pressure corresponding to the 2.6 km/s steel fragment is basically less than 0, except for a small oscillation. Compared with the steel fragment at the same speed, the pressure of the DF segment of the type I fragment is much higher. It can be seen that the DF segment is dominated by the expansion of the detonation products of the reactive material. It is evident that the detonation reaction of the reactive material enhances the duration and strength of the compressed shock wave pulse imparted to the explosive.
(2) The head of the type II fragment is covered with the steel shell; under the hypervelocity impact, the fragmentation of the internal reactive material and the activation of the detonation reaction are later than those of the type I fragment. After the reactive material is activated, the pressure generated through the expansion of the detonation products propagates to the explosive through the head shell and the charge shell in turn. Similarly to the type I fragment, the detonation products of the reactive material at the initial stage of penetration are initial expansion, and the compressive shock wave finally introduced into the explosive is dominated by the kinetic energy of the fragment, such as in the GH segment of the curve in Figure 22. Because the pressure is generated only by the kinetic energy, the compressive shock wave intensity is small at the initial stage of penetration. When the expansion of the detonation products of the reactive material is gradually strengthened, the expansion pressure is very large, but the existence of the head shell has a certain attenuation effect on the expansion pressure. Therefore, compared with the type I fragment, the duration and intensity of the shock wave pulse transmitted to the explosive are reduced, such as in the HI segment. When the detonation products expand in the opposite direction, the intensity of the compressive shock wave introduced into the explosive gradually decreases, such as in the IJ segment. Apparently, the HJ segment is dominated by the expansion of the detonation products of the reactive material. Figure 24 shows local magnification images of the penetration process of the type II fragment at typical moments in each section.

Under the impact of the 2.7 km/s steel fragment, the pressure of the explosive interface at about 13.8 μs begins to be greater than 0 and increases rapidly. The possible reason is that the explosive near observation point 2 has begun to react rapidly, and a large amount of gas products will be generated and expand to the surrounding area, so that the explosive at the interface interacts with the shell to produce high pressure. Therefore, the main influence on the detonation of explosive is the pressure pulse before 10 μs.

It can be seen from Figure 22 that at the impact velocity of 2.6 km/s, the high-pressure duration of the compression shock wave pulse imparted to the explosive and the intensity of the compression shock wave after 10 μs are in the order of the type I fragment, type II fragment, and steel fragment, from large to small. Obviously, under the impact of two composite-structure reactive fragments, the continuous long-term pulse load causes the explosion of the shielded charge. For the steel fragment at 2.7 km/s and 2.6 km/s, the shock wave pulse shape of the explosive interface before 10 μs is similar, and except that the first peak pressure corresponding to the impact velocity of 2.7 km/s is large, the pressure values at other moments are approximately equal. Therefore, the impact of the 2.7 km/s steel fragment on the shielded charge belongs to the impact-to-detonation initiation.

The threshold velocities of the two kinds of composite-structure reactive fragments are the same. The reason is that the initiation of the two types of fragments to the shielded charge is a long-term pulse load, and the influence of the type II fragment head shell under the hypervelocity impact is small. The threshold velocity of the composite-structure reactive fragments is slightly lower than that of the steel fragment. The reason is that the shielded charge shell is relatively thick. Under the action of hypervelocity impact, although the reactive material in the composite-structure fragments has a detonation reaction, because the fragments do not perforate the charge shell, most of the energy of the detonation reaction of the reactive material does not directly act on the explosive. This reduces the energy release enhancement effect of the reactive material.

**Figure 25:**
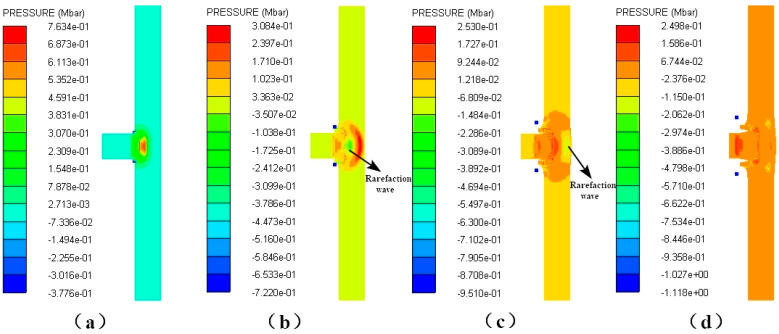
The evolution process of the rarefaction wave reflected by the interface between PTFE/Al and the head-coated shell: (**a**) 1 μs, rarefaction wave is not generated; (**b**) 2 μs, rarefaction wave has been generated and moves into the charge shell; (**c**) 3 μs, rarefaction wave moves to the explosive interface; (**d**) 4 μs, rarefaction wave enters the explosive.

As can be seen from Figure 22, there are negative pressures in all four curves. When the type II fragment impacts the shielded charge, a left-propagating shock wave is generated in the head-coated shell. When the shock wave propagates to the PTFE/Al interface, a right-propagating rarefaction wave is reflected. Since the material of the fragment-coated shell is the same as that of the charge shell, the rarefaction wave will propagate to the explosive interface, resulting in a decrease in pressure and forming a negative pressure, such as the negative pressure peak between 2.5 and 5 μs in Figure 22. The evolution process of the rarefaction wave reflected by the interface between PTFE/Al and the head-coated shell is shown in Figure 25. For the curves corresponding to the type I and steel fragments, no negative pressure is observed at 2.5–5 μs, due to the fact that they have the same medium in the main area of the head, and there are no reflected rarefaction waves. For the 2.6 km/s and 2.7 km/s steel fragments, when the shock wave reaches their left end face, the right-propagating rarefaction wave is reflected. When the rarefaction wave propagates to the explosive interface, the pressure at this place is reduced, and a negative pressure is formed. Due to the impact velocity of the 2.7 km/s steel fragment being higher, the shock wave that propagates inside it is faster. Compared with the 2.6 km/s fragment, the shock wave arrives at the left end face of the fragment earlier, and the rarefaction wave is reflected. In addition, the higher the speed, the more serious the erosion of the fragment when it penetrates the charge shell, shortening the path of the rarefaction wave propagation. Therefore, under the action of the 2.7 km/s steel fragment, the negative pressure of the explosive interface is earlier than that of the 2.6 km/s steel fragment, and the negative pressure peaks around 10 μs, as shown in Figure 22. In a way that is notably different from steel fragments, the interaction of waves in composite-structural reactive fragments is very complex at subsequent moments [34]. The pressure change at the explosive interface is also the result of this complex action. However, in any case, the energy release reaction of PTFE/Al can delay the influence of the rarefaction wave. Therefore, under the action of type I and II fragments, the negative pressure at the explosive interface appears later. From the previous analysis, it can be seen that the release energy enhancement effect of the type I fragment is high, so the negative pressure appears later than that of the type II fragment.

## 4. Conclusions

The SPH algorithm was employed in this study to simulate the hypervelocity impact of two kinds of composite-structure reactive fragments on a shielded charge with a relatively thick shell, and the results were compared with those of steel fragments possessing the same mass and diameter. For the thin-shell shielded charge, the results of this study may not be applicable to explain its impact initiation process and mechanism, and further research is needed. The principal conclusions are as follows:

(1) The threshold velocity of the impact initiation of two types of composite-structure reactive fragments on a shielded charge is 2.6 km/s, whereas the value of the steel fragment is 2.7 km/s. When the three fragments impact the shielded charge at the threshold speed, the detonation of the explosive begins at 3 cm from the interface. After detonation growth, stable detonation is finally achieved at 12 cm from the interface.
(2) At the threshold velocity, the initiation mechanism of two types of composite-structure reactive fragments and steel fragments on the shielded charge is different. The energy release reaction of the PTFE/Al material increases the time and intensity of the compression shock wave pulse in the explosive. Therefore, the hypervelocity impact of the two composite-structure reactive fragments on the shielded charge belongs to the pulse load initiation for a long time, while the steel fragment belongs to the impact-to-detonation initiation.
(3) Under the action of hypervelocity impact, when the metal shell at the front end of the composite-structure reactive fragment is thin, it has no obvious effect on the initiation of the shielded charge. For the shielded charge with a thicker shell, compared with the steel fragment, the impact initiation threshold velocity of the two composite-structure reactive fragments is not significantly reduced. The reason is that the fragments fail to perforate the charge shell, resulting in chemical energy being released by the reactive material, which does not directly act on the explosive.

## Figures and Tables

**Table 1:** The Lee–Tarver EOS parameters of the PTFE/Al material used in the simulations [22].

***ρ*/(g/cm^3^)**	***A*/GPa**	***B*/GPa**	***R*_1_**	***R*_2_**	***ω***	***D*/(km/s)**	***E*/(Gerg/mm^3^)**
2.2	15.9	0.0023	7	0.6	0.38	5.2	0.12
***P*/GPa**	***I*/μs^−1^**	***G*_1_**	***c***	***d***	***y***	***c*_0_/(km/s)**	***λ***
21	44	200	0.222	0.666	1.6	1.45	2.25

**Table 2:** The strength model parameters of the PTFE/Al material used in the simulations [22].

*A*/MPa	*B*/MPa	*n*	*C*	*m*	*T_m_/*K	*T_r_/*K
48	64.1	0.574	0.219	0.226	653	300

**Table 3:** The material parameters of 4340 steel used in the simulations [27,28].

*ρ*/(g/cm^3^)	*c*_0_/(km/s)	*λ*	*A*/MPa	*B*/MPa	*n*	*C*	*m*	*T_m_/*K
7.823	4.57	1.49	792	510	0.26	0.014	1.03	1793

## Data Availability

Data are contained within the article.
